# You Were Always on My Mind: Introducing Chef’s Hat and COPPER for Personalized Reinforcement Learning

**DOI:** 10.3389/frobt.2021.669990

**Published:** 2021-07-16

**Authors:** Pablo Barros, Anne C. Bloem, Inge M. Hootsmans, Lena M. Opheij, Romain H. A. Toebosch, Emilia Barakova, Alessandra Sciutti

**Affiliations:** ^1^ Cognitive Architecture for Collaborative Technologies (CONTACT) Unit Istituto Italiano di Tecnologia, Genova, Italy; ^2^ Department of Industrial Design, University of Technology Eindhoven, Eindhoven, Netherlands

**Keywords:** continual reinforcement learning, human modeling through online games, opponent-aware competitive learning, simulation environment for reinforcement learning, reinforcement learning

## Abstract

Reinforcement learning simulation environments pose an important experimental test bed and facilitate data collection for developing AI-based robot applications. Most of them, however, focus on single-agent tasks, which limits their application to the development of social agents. This study proposes the Chef’s Hat simulation environment, which implements a multi-agent competitive card game that is a complete reproduction of the homonymous board game, designed to provoke competitive strategies in humans and emotional responses. The game was shown to be ideal for developing personalized reinforcement learning, in an online learning closed-loop scenario, as its state representation is extremely dynamic and directly related to each of the opponent’s actions. To adapt current reinforcement learning agents to this scenario, we also developed the COmPetitive Prioritized Experience Replay (COPPER) algorithm. With the help of COPPER and the Chef’s Hat simulation environment, we evaluated the following: (1) 12 experimental learning agents, trained *via* four different regimens (self-play, play against a naive baseline, PER, or COPPER) with three algorithms based on different state-of-the-art learning paradigms (PPO, DQN, and ACER), and two “dummy” baseline agents that take random actions, (2) the performance difference between COPPER and PER agents trained using the PPO algorithm and playing against different agents (PPO, DQN, and ACER) or all DQN agents, and (3) human performance when playing against two different collections of agents. Our experiments demonstrate that COPPER helps agents learn to adapt to different types of opponents, improving the performance when compared to off-line learning models. An additional contribution of the study is the formalization of the Chef’s Hat competitive game and the implementation of the Chef’s Hat Player Club, a collection of trained and assessed agents as an enabler for embedding human competitive strategies in social continual and competitive reinforcement learning.

## 1 Introduction

Modeling competitive and cooperative behavior as a continual adaptation mechanism is one of the most important and challenging goals of human–robot interaction (Crossman et al., 2018). The tasks that social robots are expected to perform in the near future demand not only effective perception of social cues but also the understanding of intentions and contextual interactions, along with humanlike decision-making. In particular, the development of cognitive architectures to deal with social interactions has become of great interest in recent years (Franklin et al., 2013; Sandini et al., 2018; Gorbunov et al., 2019; Tanevska et al., 2020). Social interactions are highly complex and to allow fluent and natural cooperation with humans, artificial agents must take into consideration the continual and dynamic aspects of human social behavior. Providing the proper response, which sometimes needs to be what the partner expects and sometimes needs to be novel and interesting, is one of the most important measures to achieve a natural engagement with an artificial agent (Hirokawa et al., 2018).

A common problem, however, arises when developing social agent behaviors that resemble the richness of human interaction and decision-making. To design, evaluate, and validate such systems in a lifelong learning scenario is an expensive task, even in closed-loop scenarios, since many examples from different persons behaving in different manners and situations are needed. This usually makes such scenarios not reproducible or challenging to evaluate in long-term performance. Due to this limitation, most of the current solutions for social cognitive architectures in robots are based on interaction strategies focused on one-time simple decision-making (Van de Perre et al., 2018) or rely upon simple decision trees for generating somehow expected behaviors based on a single-task observation space (Tuyen et al., 2018).

Games are shown to be a useful tool for restricting the number and type of interactions while still providing a richness of interaction possibilities more similar to that encountered in the real world. To address the problem of providing a standard and easily reproducible online interaction scenario for the development of artificial social agents, we developed and validated a novel card game (Barros et al., 2021). The game, named Chef’s Hat, was designed to be played by four subjects and contains a complex strategy formation that is directly affected by how the opponents play the game. The entire game-flow was designed to be easily adapted to artificial agents, without breaking the natural interaction observed when humans play it.

In a recent investigation (Barros et al., 2020), we demonstrated that learning agents can play the game and present a good performance, measured as number of victories, when playing against each other. We observed that each agent learned a different gameplay style, based on the learning algorithm each of them implements. All the evaluated agents, however, learn in an off-line manner, which reduces their applicability in a real-world scenario and replicates what has been shown in most of the recently proposed simulation environments.

In this study, we extend and formalize the online learning version of the Chef’s Hat simulation environment. To validate it, we introduce COmPetitive Prioritized Experience Replay (COPPER) to provide reinforcement learning agents with the ability to learn and adapt to other opponents continuously. COPPER implements another weight on the prioritized experience replay algorithm, relating specific experiences with specific opponents. Moreover, COPPER is a continual learning algorithm (Khetarpal et al.,) but can be distinguished as a form of “personalized” learning.

Our experiments aim to demonstrate how our online learning agents compare with the existing off-line Chef’s Hat players (Barros et al., 2020) and in a smaller scale with human players, by using an extension of the environment that allows humans to play against the trained agents. We extend three agents, based on deep Q-learning (DQL) (Mnih et al., 2013), proximal policy optimization (PPO) (Schulman et al., 2017), and actor-critic with experience replay (ACER) (Wang et al., 2016), to learn online how to play the game. We implement 12 versions of off-line and online agents in total to better explain the contribution of COPPER to competitive reinforcement learning. This way, we made these agents create personalized strategies to beat their opponents.

Our entire evaluation is based on a tournament scenario where all the developed agents play several rounds of Chef’s Hat against each other. In our analysis, we evaluate the behavior of the agents in terms of the number of victories and track the performance over the number of played games. We discuss how the online learning agents can adapt and develop a fast-paced strategy-learning behavior when playing against the off-line learning agents. We also analyze the role of COPPER in adapting faster to an opponent’s strategy. Finally, in our small-scaled human experiment, we are able to discuss how there is a trend that demonstrates that the performance of the online learning agents is better when playing against humans than that of the off-line trained agents.

As a contribution of this study, first, we introduce the formalization of a reproducible and challenging simulation scenario for multiple agents. Second, we provide the Chef’s Hat Players Club as a collection of implemented, ready-to-use, and evaluated agents for Chef’s Hat with the hope to facilitate the future development of this environment and, moreover, to facilitate the advancement of the research on online and personalized competitive learning in social contexts. Third, we propose the novel COmPetitive Prioritized Experience Replay (COPPER) algorithm and analyze it in a competitive multiplayer continual learning scenario, showing its advantages in comparison to the Prioritized Experience Replay (PER) algorithm.

## 2 Related Work

The limitation on designing, implementing, and evaluating realistic interaction scenarios, in particular where multiple people are involved and perform a lasting interaction, is one of the problems that must be solved before the deployment of artificial agents, such as robots, in real life. Robots do not have universal skills yet, so most of the common interaction scenarios provide a restricted action space. To overcome this limitation, several human–robot– and human–agent–based scenarios are developed as games, as they have been proven to capture some relevant aspects of natural group interaction. For instance, a game specially designed to favor one player and disfavor another player in a multiplayer interaction was shown to provoke emotional reactions (Barakova et al., 2015), which lasted during the repetitive games between the same players (Gorbunov et al., 2017).

With the recent development of reinforcement learning algorithms, the design and implementation of adaptable simulation environments flourished (Brockman et al., 2016). Such environments allow fast-paced simulations of different tasks and the calculation of specific step-reward functions and are the basis for the recent groundbreaking applications of deep reinforcement learning. Most of these scenarios, however, are not developed to be used in continual or online learning tasks, nor on social interactions, as they usually focus on optimizing one single task. Most of them simulate situations that are based on single agents (Shi et al., 2019), classic reinforcement learning problems (Cullen et al., 2018), robotic simulations (Zamora et al., 2016), or, more recently, playing video games (Torrado et al., 2018).

Although there has been a recent interest in continual and online reinforcement learning (Lomonaco et al., 2020), most of these applications and scenarios take into consideration single agents or nonsocial interactions (Khetarpal et al., 2020). The development of online reinforcement learning toward social applications, in particular competitive environments, is yet to be largely explored, although there exists a relevant effort on multi-agent investigation (Nekoei et al., 2021). The focus on generalization makes these solutions ideal for multitask learning but hinders them from developing personalized strategies when facing multiple opponents in a competitive game, for example.

To evaluate online and competitive reinforcement learning in real-world scenarios demands a strong and adaptable simulation environment. Usually, this is achieved with game simulations, where the decisions of an agent impact their future behavior. These environments, however, mostly focus on single-agent tasks or, at maximum, dyadic interactions. A simulation environment that relies on online learning and provides a multi-agent task is still not easily available for the general public. We envision that with the formalization of the Chef’s Hat environment, we can fill this gap.

The lack of such an environment, however, did not stop the study of online reinforcement learning, and many researchers recently proposed different solutions for it (Lillicrap et al., 2015; Zhang et al., 2018). These range from transfer of learned representations to constituting a lifelong learning system (Abel et al., 2018), the development of a focused experience replay (Schaul et al., 2015) for tuning a policy network toward adapting to new environments (Ye et al., 2020), or achieving scalable multitask learning with several workers (Zhan et al., 2017). Although these solutions seem to work well in complex tasks, most of them demand a higher amount of training loops than off-line reinforcement learning, which makes them not viable in a human–artificial agent interaction scenario. Thus, in this study, we propose a simple adaptation of a prioritized experience replay to reduce the demand of many training loops but still leverage the advantage of online learning through personalization.

## 3 The Chef’s Hat Simulation Environment

The scenario that we propose is based on a card game played by four players. The card game scenario was chosen as the underlying action–perception cycle development environment as it represents a controllable situation, where each player has its turn to take specific actions and yet provides a ground for natural interaction between the players. Within this scope, we needed a game that allows the players to develop strategies while playing and that, within the game mechanics, evoked a dynamic competitive behavior.

**FIGURE 1:**
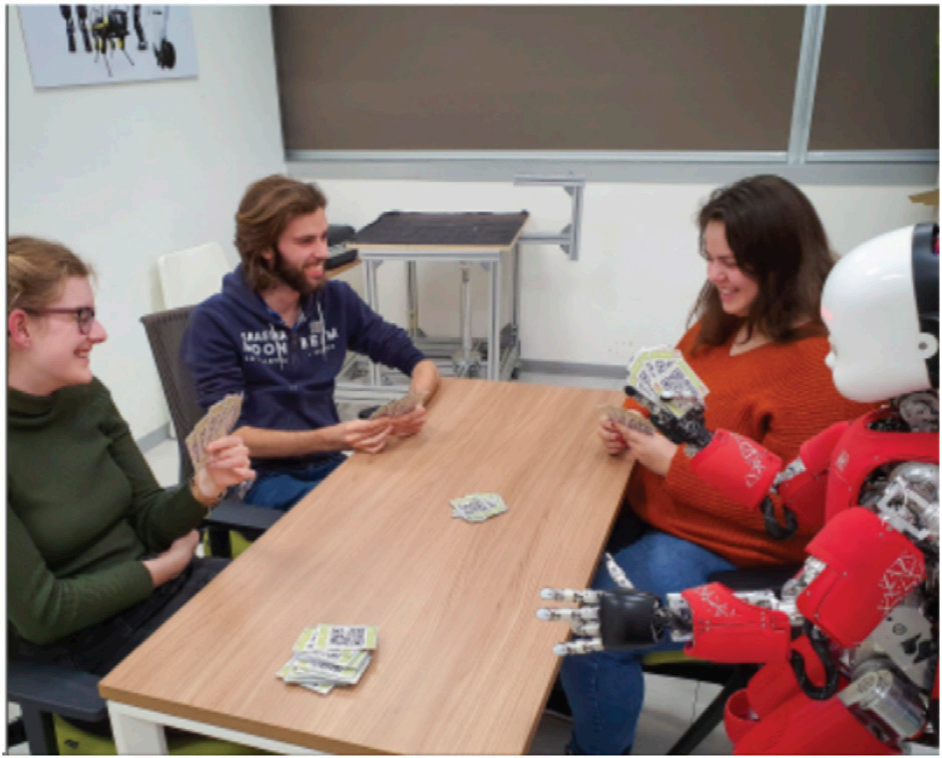
Illustration of the Chef’s Hat card game being played by three humans and a robot.

In this regard, we recently developed and validated the Chef’s Hat card game (Barros et al., 2021), illustrated in Figure 1. In this study, we propose a 1:1 implementation of the game in an OpenAI-like gym environment (Brockman et al., 2016). Below, we describe the game mechanics and the details of the environment functionalities.

### 3.1 Embedded Chef’s Hat Mechanics

The development of the Chef’s Hat mechanics followed two main principles: 1) to provide restricted, but natural, competitive interaction between the four players and 2) to provide turn-taking, that is, an organized structure, in which an agent has a supportive infrastructure and capacity to process incoming information and generate behavior without breaking the fluidity of the interaction.

The game simulates a kitchen environment, and it has a role-based hierarchy: each player can either be a Chef, a Sous-Chef, a Waiter, or a Dishwasher. The players try to be the first to get rid of their ingredient cards and become the Chef. This happens for multiple rounds (or Shifts, each of them detailed in **Algorithm 1**) until the first player reaches 15 points.

As exhibited in **Algorithm 1**, during every Shift there are three phases: Start of the Shift, Making Pizzas, and End of the Shift.

At the Start of the Shift, the cards are shuffled and dealt by the players. Then, the exchange of roles starts based on the previous Shift end positions. Whoever finished first becomes the Chef, whoever finishes second becomes the Sous-Chef, third the Waiter, and fourth the Dishwasher. The change of roles is necessary to change the game balance, rewarding the players who finished first in the last Shift and encouraging them to win the next one. Once the roles are exchanged, the players have the chance to do a special action. If a player has two jokers at the start of the Shift in their hand, they can choose to play their special action: in the case of the Dishwasher, this is Food Fight (the hierarchy is inverted), and in the case of the other roles, it is Dinner is served (there will be no card exchange during the Shift). Then, unless the action “Dinner is served” is played, the exchange of the cards starts. The Dishwasher has to give the two cards with the highest values to the Chef, who in return gives back two cards of their liking. The Waiter has to give their lowest-valued card to the Sous-Chef, who in return gives one card of their liking.

Then, the Making of the Pizzas starts. The person who possesses a “Golden 11” card may start making the first pizza of the Shift. A pizza is prepared when ingredient cards are played on the pizza base on the playing field. A pizza is made when no one can (or wants to) put on any ingredients anymore. The rarest cards have the lowest numbers. A card can be played if it is rarer (i.e., lower face values) than the previously played cards. The ingredients are played from the highest to the lowest number, so from 11 to 1. Players can play multiple copies of an ingredient at once but always have to play an equal or greater amount of copies than the previous player did. If a player cannot (or does not want to) play, they pass until the next pizza starts. A joker card is also available, and when played together with other cards, it assumes their value. When played alone, the joker has the highest face value (12).

**Algorithm 1** The playing flow of one Shift of the Chef’s Hat card game.


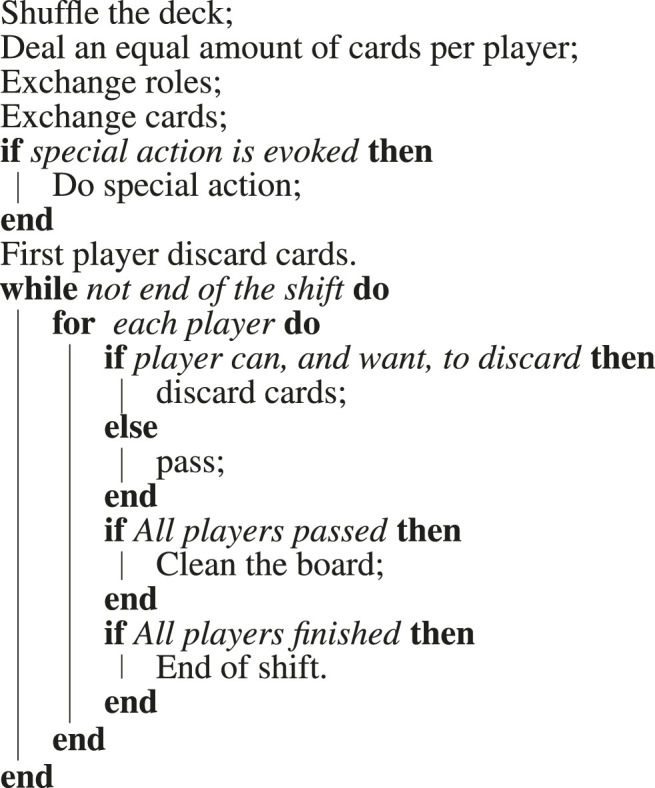


At the end of the Shift, the new roles are distributed among the players according to the order of finishing, and every player gets the number of points related to their role. The Chef gets five points, the Sous-Chef gets three points, the Waiter gets one point, and the Dishwasher gets 0 points. The game continues until one of the players reaches 15 points.

**FIGURE 2:**
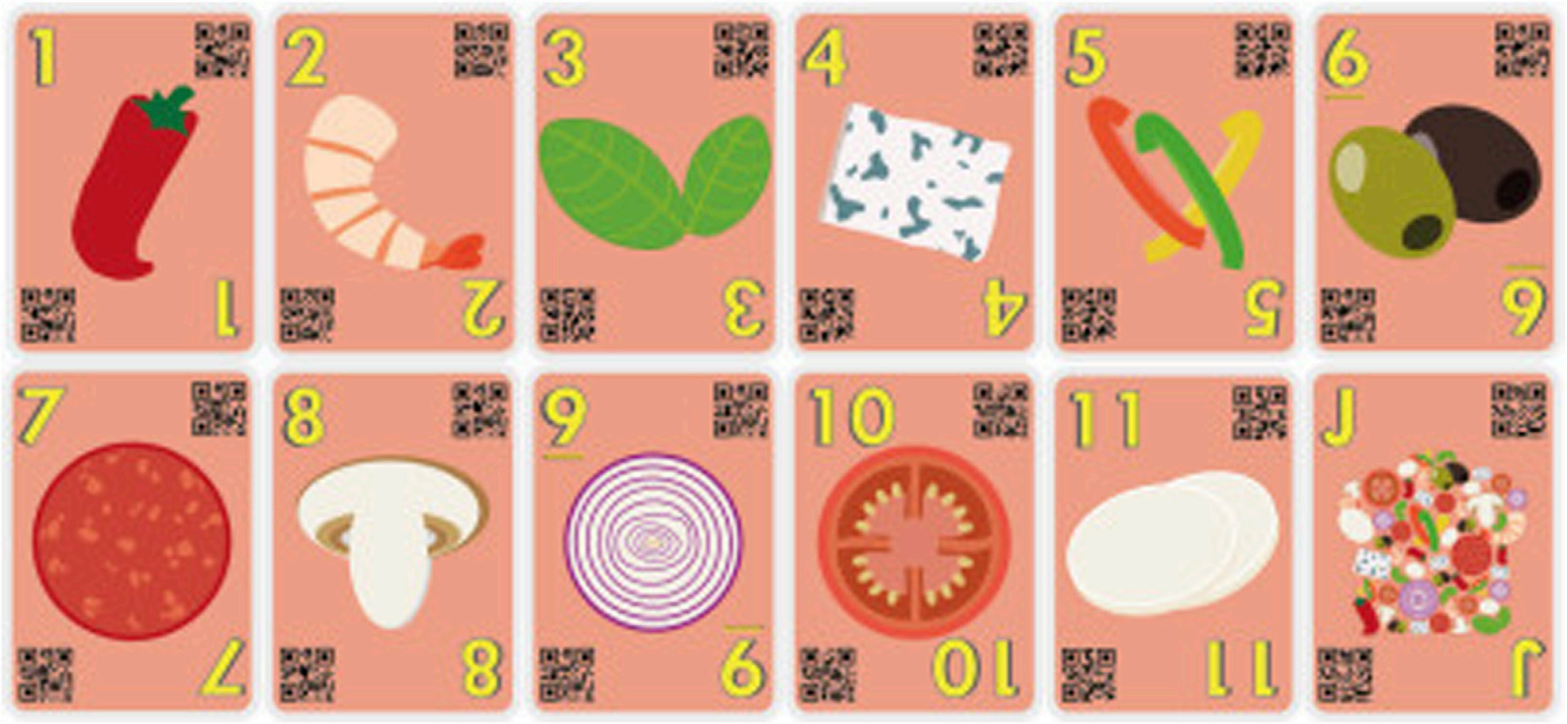
Ingredient cards and the joker, with their corresponding face numbers. The lower the number, the rarer the card.

The ingredient cards, illustrated in Figure 2, needed to be easily recognizable by all potential players, including humans and robots, both when played on the playing field and when exchanged among players at the start of the Shift. The pictures in the cards are easy to recognize for human players, and to ease the recognition by the robots, QR-codes were added. The QR-codes allow a camera placed on the top of the playing field to capture the overall game state and save it. This is extremely important when creating a learning database to be used together with the virtual environment.

**FIGURE 3:**
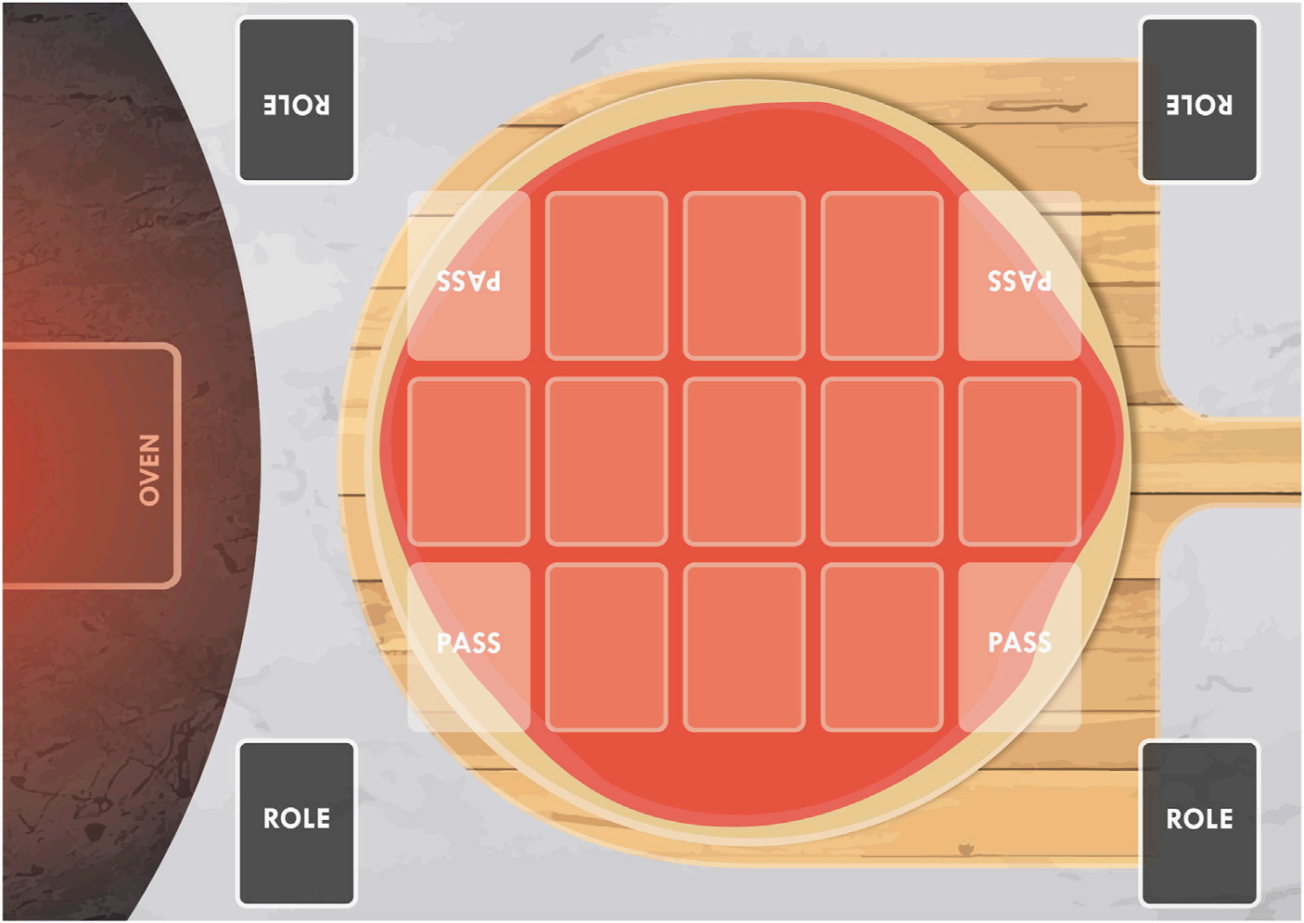
Playing field where the cards are placed, representing a pizza board.

The cards are to be placed on the playing field, illustrated in Figure 3. To guarantee that the players lay down the cards without stacking them and hindering their recognition by automatic systems, we designed the playing field to have 11 different marked places in which players could place their cards on the pizza.

### 3.2 OpenAI Gym–Based Environment

OpenAI Gym Brockman et al. (2016) is a very popular toolkit that facilitates the development and the dissemination of simulation environments for training reinforcement learning agents. It enables the creation of standardized environments that allow the establishment of a set of specific rules for a simulation, the calculation of varied types of rewards, and the logging and visualization of training artificial agents. Recently, several simulation environments were released using the OpenAI Gym, which facilitates its reproduction and evaluation with different reinforcement learning algorithms.

We ported the Chef’s Hat game into the OpenAI Gym and implemented all the complex game rules and mechanics. The environment is freely available,^1^ and we envision that this environment will help to standardize the learning of game strategies within our card game but also to collect and share data for different reinforcement learning–based players.

The environment calculates the game state by aggregating the current player’s hand and the current cards on the board. Using this standard state representation, we can give the learning agents the possibility to learn specific strategies purely based on the cards they hold and the cards which were displayed. Of course, as the environment is fully customizable, the current game state can be composed of any other variable which might help the agent to succeed in their task.

Each action taken by an agent is validated based on a lookup table, created on the fly, based on the player’s hand and the cards in the current playing field, to guarantee that a taken action is allowed given the game context. The lookup table is extremely important for games when humans are involved as it guarantees that the game rules are maintained.

**FIGURE 4:**
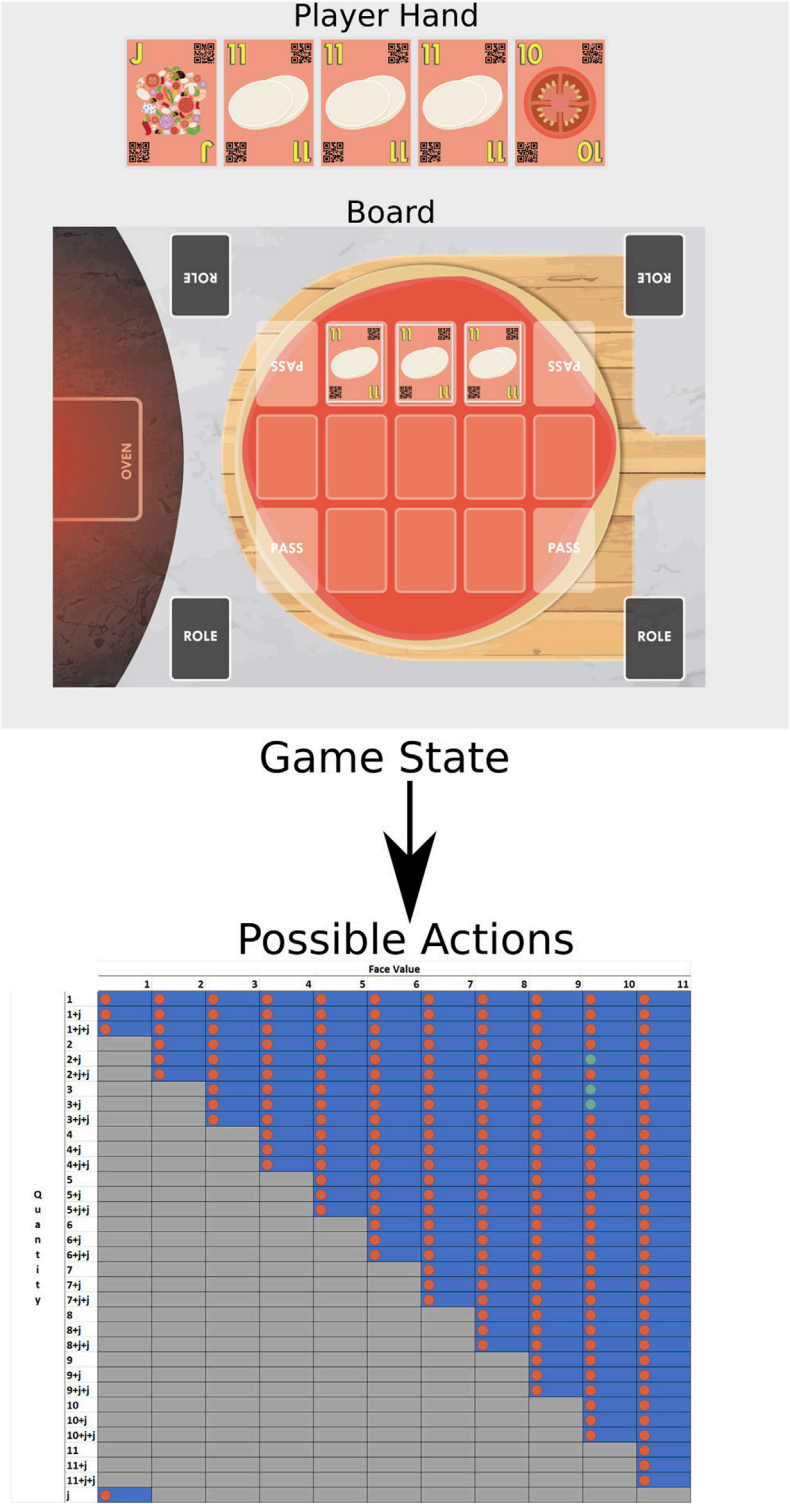
Example of possible actions given a certain game state. The columns represent the card face values, and the rows represent the number of cards to be discarded. The letter “j” represents the presence of a joker. The lookup table is created on the fly, and it marks all the actions which are allowed based on the game mechanics (blue regions) and the ones which are not allowed (gray regions). For this given game state, the player would be allowed to only perform the actions marked with the green dots.

The actions are calculated based on the number of possibilities of the lookup table. The standard game, with a deck of 121 cards, has a total of 200 possible actions, which capture all the possible moves a player can make: to discard one card of face value 1 represents one move and to discard 3 cards of face value 1 and a joker is another move, while passing is considered as yet another move. Figure 4 illustrates an example of calculated possible actions given a game state. The blue areas mark all the possible action states, while the gray areas mark actions that are not allowed due to the game’s mechanics. We observed that given this particular game state, a player would only be allowed to perform one of three actions (marked in green), while any other action (marked in red) would be considered as invalid and not carried out.

The environment allows the customization of the game itself. We can easily choose how many players will be playing the game, how many cards a deck can have, and how many of the playing agents are to be trained. The agents are also customized and follow a standard implementation protocol. This allows the implementation and deployment of a large variety of agents, from complex learning agents that might take external factors to learn the game strategy (e.g., from an external camera reading a real game) to naive agents that do specific actions following simple rules.

For each action that an agent performs, the game environment calculates a specific reward. Again, as the environment is fully customizable, the reward calculation can be updated according to the needs of the training agents. For example, giving the highest reward for an agent that performs a valid action, that is, an action that follows the game rules, can be used to train an agent to learn the rules of the game. Later on, this reward can be updated to make the agent learn how to win the game.

Another aspect of our environment is the logging of actions and states. It allows us to create snapshots of each played game, which can be used to create playing datasets which are extremely helpful for further training intelligent agents. Each step of the gameplay is recorded in a different set of files and can be retrieved later on with ease.

The stored games can be used by the environment as a modulation for specific agents to behave in a particular manner. That allows information obtained from real-world games, collected while real persons are playing them, to be easily inserted into the environment as primitives for the training of the agents. The game status of the real games can be obtained *via* a single camera facing the playing field and when saved in the same format as the one used by the environment, can be imported and used during the game.

### 3.3 The Chef’s Hat Online

**FIGURE 5:**
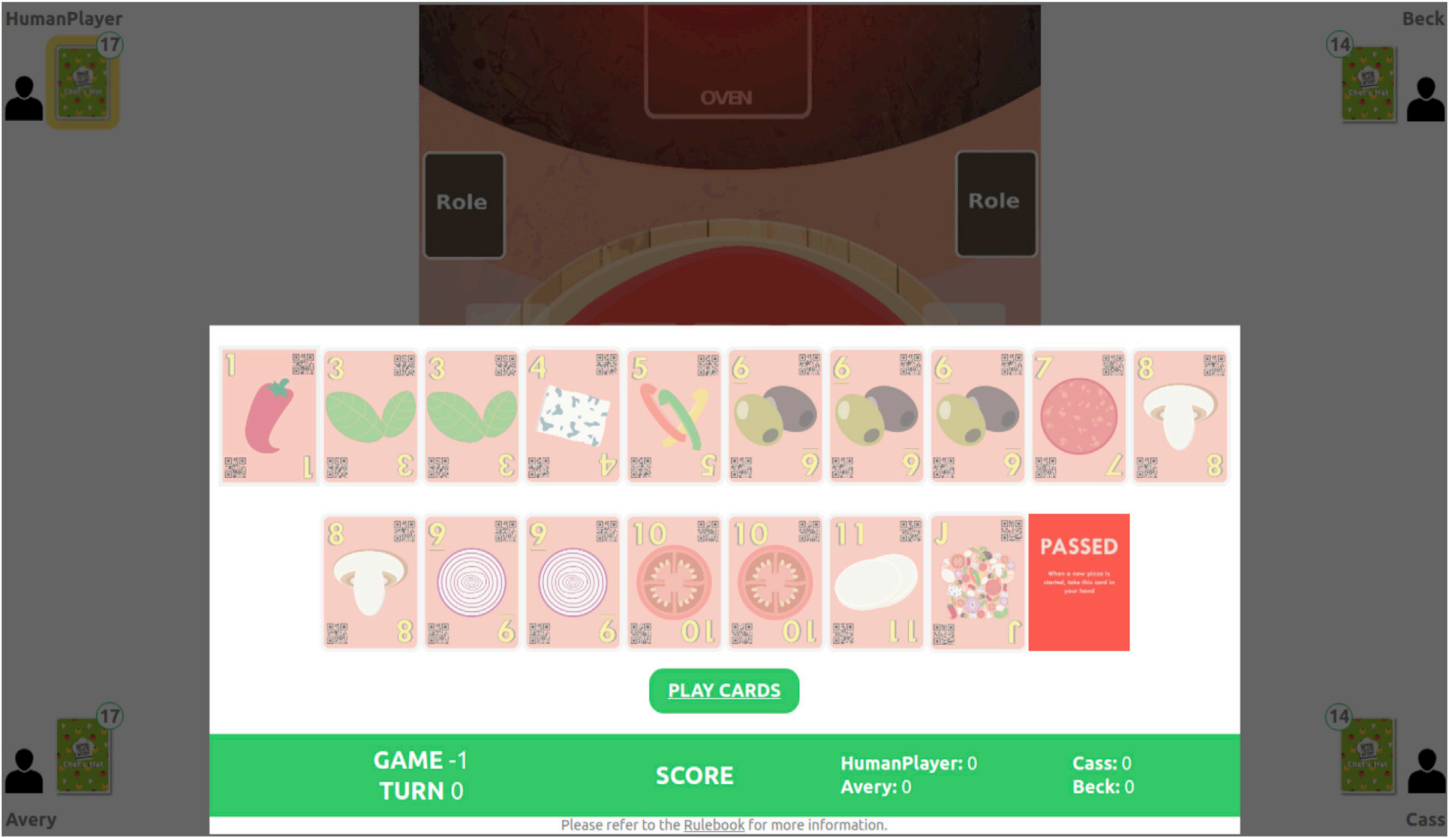
Chef’s Hat Online interface allows humans to play against trained agents. This solution allows the collection and exporting of the entire gameplay in a format compatible with the Chef’s Hat Gym environment.

In order to facilitate the integration and evaluation of the developed agents in a real-world scenario, we depict the Chef’s Hat environment in the Chef’s Hat Online game^2^, illustrated in Figure 5.

Chef’s Hat Online is a web-based interface that allows experimenters to set up a game where a human can play against three different agents. The game follows the same rules as the physical game and has an interface completely adapted for web-based interaction. It collects all the information the Gym-based environment does and saves it in the same format, allowing the use of all the logging and plotting to generate tools already present in the Gym-based environment.

## 4 The Chef’s Hat Competitive Learning Agents

The general Q-learning algorithm learns to maximize the probability of choosing an action that leads to maximum reward. For that, it calculates a Q-value (quality value) for each action given a state and updates the policy, in our case represented by a neural network, to maximize the expected reward. Using a temporal difference calculation, it can take into consideration a sequence of steps that leads to the final state. In our simulation environment, the final state is achieved when the player has no cards left in their hand. The maximal reward is gained once the player is the first one to reach the final state.

### 4.1 Defining Chef’s Hat Q-Learning

The typical Q-learning algorithm represents a function *Q* as follows:

Q:SxA→ℝ, (1)

where *S* is the state, in our case represented by the 28 values composed by the cards at hand and the cards at the board. The actions, *A*, are expressed using the 200 discrete values for all the possible actions.

To update the Q-values, the algorithm uses the following update function:

$$Q^{\prime }\left(s_{t},a_{t}\right)=Q\left(s,a\right)+\alpha \times \left(TD\right),$$ (2)

where *t* is the current step, α is a predefined learning rate, and TD is the temporal difference function, calculated as follows:

$$TD=r_{t}\times \gamma \times maxQ\left(s_{t+1,a_{t}}\right)-maxQ\left(s_{t+1,a_{t}}\right),$$ (3)

where $r_{t}$ is the obtained reward for the state ($s_{t}$) and action ($a_{t}$) association, γ represents the discount factor, a modulator that estimates the importance of the future rewards, and $maxQ\left(s_{t+1,a_{t}}\right)$ is the estimate of the Q-value for the next state.

To improve the capability of Q-learning algorithms, experience replay is used to store the agent’s own experience and use it to improve learning. It saves important steps taken by the agents to increase the available data for learning state/action pairs through batch-learning.

### 4.2 Competitive Prioritized Experience Replay

When applied to online learning problems, Q-learning–based reinforcement learning usually presents suboptimal results. The problem is even more critical when applied to multi-agent competitive scenarios, where an agent has to counter the opponent’s actions. To achieve an optimal generalized behavior, the agent must acquire a large enough experience, which usually takes time and a high number of experiences. In the proposed scenario, the use of stored experiences biases the agents to understand that all the adversaries play the game similarly to each other. In a competitive scenario, this is usually not the case.

Prioritized experience replay (PER) (Schaul et al., 2015) can help us handle the online update aspect of competitive learning. Originally, experience replay adds a weighted probability [P(i)] on the recorded experience pool, so that each experience has a different meaning when it is used to update the Q-values. P(i) is calculated based on the network’s loss after calculating TD in a forward pass of the network (using an input *i*) as follows:

$$P\left(i\right)=\frac{p_{a}^{i}}{\sum_{k}p_{a}^{k}},$$ (4)

where *a* indicates how much we want to rely on the priority, *p* is the priority, and *k* is the total number of saved experiences. It lacks, however, the ability to individualize the learning toward a specific opponent. So every time an agent learns using PER, it pulls from the most successful previous experiences and iterates over them, updating its knowledge. This creates a generally good agent, but it is unable to adapt to specific opponents quickly. To deal with that, we update PER by adding an individualized term.

As Chef’s Hat is a multiplayer competitive game, our experience replay adds another piece of information to be weighted, which is the relevance of this experience when playing against that specific opponent (*o*), as shown below:

$$P\left(i\right)=o\frac{p_{a}^{i}}{\sum_{k}p_{a}^{k}}.$$ (5)

We update *o* based on the relative performance of the agent in comparison with those of the opponents at the end of each game, so if the agent was better than the opponent, these experiences have a higher impact.

## 5 Evaluating Online Learning Agents on Chef’s Hat

To best evaluate our COPPER-based agents, we first establish a set of opponents, the Chef’s Hat Players Club, and the tournament scenario. Each of our experiments uses agents from the Players Club and investigates different aspects of COPPER-based agents.

### 5.1 Chef’s Hat Players Club

To provide a general understanding of the impact of our proposed competitive continual learning agents on the Chef’s Hat environment, we introduce here the Chef’s Hat Players Club^3^—a collection of implemented and optimized agents for Chef’s Hat.

First, we implement two dummy agents: one that performs random movements ($D_{r}$) and one that only discards one card at a time ($D_{o}$). Then, we developed versions of agents based on deep Q-learning (DQL) (Mnih et al., 2013), proximal policy optimization (PPO) (Schulman et al., 2017), and actor-critic with experience replay (ACER) (Wang et al., 2016). These are the most popular, and effective, reinforcement learning algorithms for game scenarios.

In our previous study (Barros et al., 2020), we proposed two different manners to train these agents off-line on Chef’s Hat: make them play against the naive agents (*off*_*n*_) or against different generations of themselves in a self-play strategy (*off*_*s*_). In the experiments reported in this article, we will use both variations.

For online learning, we will implement our competitive prioritized experience replay for the three agents ($on_{c}$). Also, we implement versions of them with the traditional experience replay ($on_{e}$), to better understand the impact of the proposed solution.

In total, the Chef’s Hat Players Club is composed of 14 different agents, summarized in Table 1.

**TABLE 1:** Structure of the Chef’s Hat Players Club containing a total of 14 different agents: two dummy agents and 12 learning agents, each of them implementing the listed learning strategies with DQL, PPO, and ACER.

Name	Type	Training	Strategy
$D_{r}$	Dummy	–	Random
$D_{o}$	Dummy	–	Discard one card
*off*_*s*_	Learning	Off-line	Self-play
*off*_*n*_	Learning	Off-line	Versus naive
$on_{c}$	Learning	Online	COPPER
$on_{e}$	Learning	Online	PER

The Supplementary Material to this article contains a detailed explanation of how each of the agents was trained and optimized to play the game. It also contains a detailed description of the final architecture of each agent.

### 5.2 Tournament Scenario

To provide an experimental setup that helps us to better understand the contributions of the proposed COPPER-based agents without requiring an enormous set of combinatorial experiments, our main evaluation happens in the form of a tournament among all the agents on the Chef’s Hat Players Club.

Each tournament is composed of two brackets of hierarchical playing phases. In each phase, the agents will face each other, and the two victorious ones will advance to the next phase and play against themselves. The best agents of each bracket play against each other, and the victorious agent is crowned the winner of the tournament.

To properly assess each of the types of learning agents, we implement one instance of each of them. To complement the agents in order to allow the tournament to happen, we implement an equal number of dummy agents until we have a total of 32 agents. In our experiments, we have 3 phases per bracket and a final game to crown the champion.

### 5.3 Experimental Setup

Our experimental scenario serves as our ablation study and baseline and is the basis for our discussion and analyses. On it, we run 10 tournaments in a row, which allow the online learning agents to learn competitive interaction. We repeat this experiment 1,000 times.

In our analysis, first, we compute and analyze the average number of victories per agent and per tournament run. This informs us of the performance of each agent, giving us the differences between off-line and online agents and between the PER- and COPPER-based agents.

Our second experiment focuses on examining the differences between COPPER and PER when playing against different types of agents. We create two game setups: one where the online learning agent plays 10 games in a row against three types of different agents ($PPO_{off_{s}}$, $DQL_{off_{s}}$, and $ACER_{off_{s}}$) and the second one where the online learning agent plays 10 games in a row against the same types of agents (three instances of $DQL_{off_{s}}$). To simplify our analysis and to focus on explaining the differences between COPPER and PER, the online learning agents will be implemented using only one type of learning algorithm, $PPO_{on_{e}}$ and $PPO_{on_{c}}$.

In the third experiment, humans play against the agents using the Chef’s Hat Online variant of the game. As playing with humans is a costly task (in terms of time and effort required to organize and collect participants) and since our goal is to investigate the differences between off-line and online learning and between PER-based and COPPER-based agents, we simplify this experimental setup in terms of evaluated agents. We asked 10 human players to play 10 games against two sets of agents: the first composed of $PPO_{off_{s}}$, $PPO_{off_{n}}$, and $D_{r}$ and the second composed of $PPO_{on_{c}}$, $PPO_{on_{e}}$, and $PPO_{off_{s}}$. The PPO implementations were chosen because they are the ones with the best performance in general when compared to the other two reinforcement learning algorithms. We calculate the average number of victories in each scenario. The first set of experiments will help us to establish the off-line benchmark, while the second one will illustrate the differences between online and off-line agents, in terms of performance, and between COPPER-based and PER-based online learning.

## 6 Results

### 6.1 Tournament Results

**FIGURE 6:**
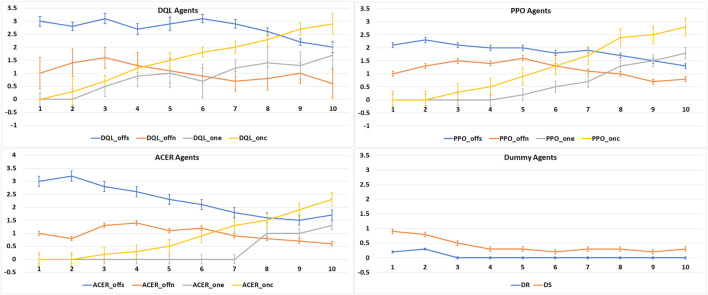
Average number of victories (Y-axis) for 1,000 runs of 10 tournaments (X-axis) in a row per agent type.

Our first experimental results, the average number of victories per tournament, are displayed in Figure 6, together with their standard deviation. By plotting these results per agent type, we can visualize the impact of online learning in each of the reinforcement learning algorithms. For all three learning agent types, the $DQL_{off_{s}}$ variation starts with the highest average number of victories in the first games, as was expected based on our previous results (Barros et al., 2020). However, after just a couple of games, the online learning agents start to increase their number of victories and in all three types of reinforcement learning algorithms, overcome the off-line learning strategies.

When compared with each other, we see that the COPPER-based agents exhibit a more steep learning curve, which shows that the proposed competitive version of the prioritized experience replay enhances the learning of specific strategy winning patterns when playing against different agents. Also, we see that the COPPER-based agents reach a higher average number of victories, without any error overlap, showing that besides a stable learning curve, they also learned more effective strategies at the end of the 10 tournaments.

### 6.2 COPPER *vs.* PER

**FIGURE 7:**
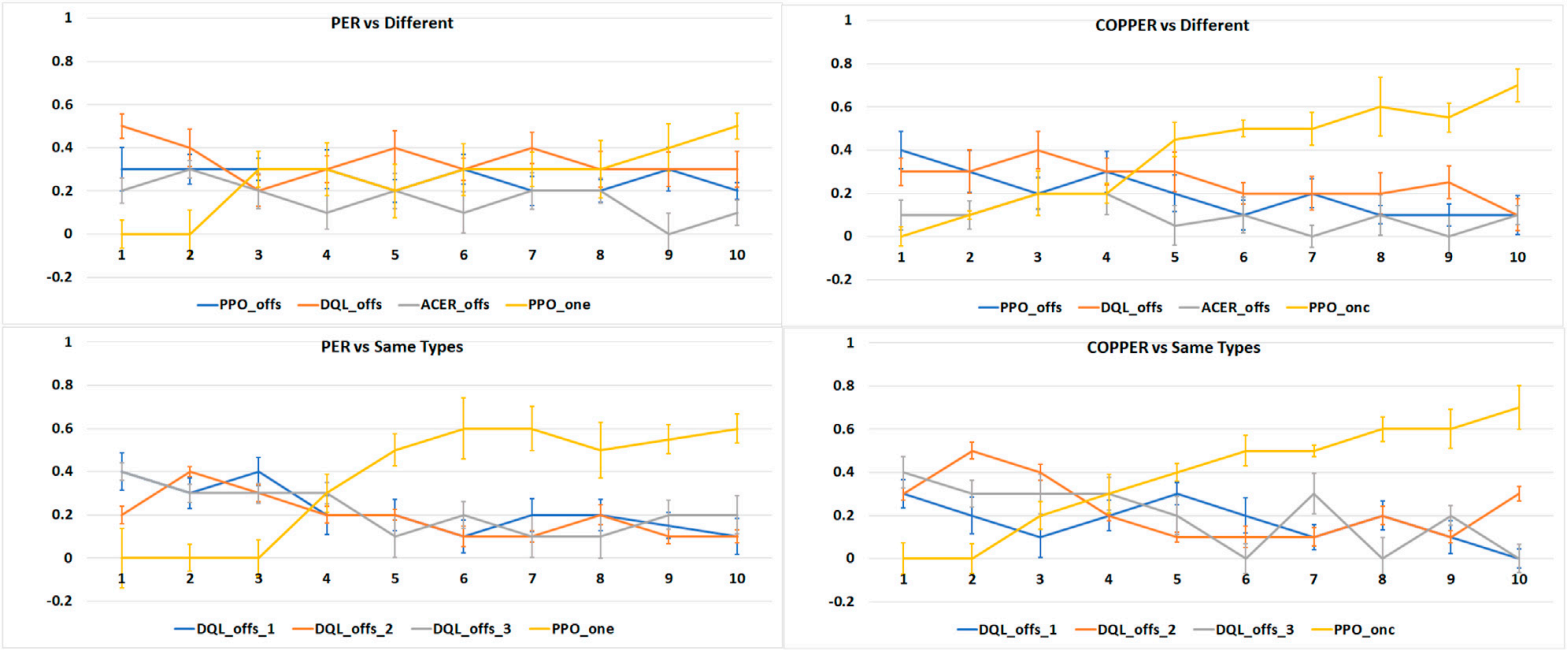
Average number of victories (Y-axis), in 1,000 runs of 10 tournaments (X-axis), per experiment type: online learning agents vs. the same types of agents and online learning agents vs. different types of agents.

Analyzing the results of our second experiment in Figure 7, where we create games that have $PPO_{on_{e}}$ and $PPO_{on_{c}}$ playing against a group of the same type of and different agents, we observe the advantage of the online learning methods. In all scenarios, the online learning agents present the best performance, in terms of average victories, by the end of the 10 games’ run.

We can also observe that COPPER achieves a higher number of victories in fewer games than the PER agents. As COPPER has specific weights on the experience replay per type of opponent, it learns how to adapt to these specific opponents’ strategies, while PER relies on a general experience pool, and thus, tries to create a general, and in this case more ineffective, strategy.

When playing against the same type of agents, the behavior of COPPER and PER is similar, which is expected as there is no specific weight attribution to the replay pool. Thus, the agents learn using the same algorithm.

### 6.3 Human on the Loop

**FIGURE 8:**
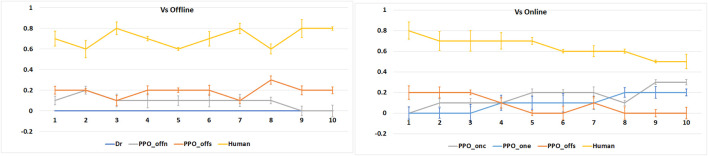
Average number of victories (Y-axis), for 10 human players, per agent type when playing 10 games in a row (X-axis).

When playing against a human, we observe (plotted in Figure 8) that none of the agents is particularly effective. We see, however, that when playing against the online learning agents, human performance drops toward the end of the game, indicating that the online agents’ performance increases. We also see that the COPPER-based agent achieved the best average victories when compared with the other agents.

## 7 Discussions

Our experiments demonstrate the main advantages of the COPPER algorithm and, with this, the advantages that come from using dynamic and online adaptation in multiple agents’ contexts. First, COPPER improves the performance of online reinforcement learning in the Chef’s Hat game. Second, the proposed three experimental setups help us to quantify the degree of these improvements. However, numbers and benchmarking are not the main contributions of this study; rather, the intended contribution is summarized in the following subsections.

### 7.1 Chef’s Hat Simulation Environment

In this article, we describe the implementation, the evaluation, and the experimental testing of a multiplayer competitive game that involves elements of reinforcement learning that are suitable for serial play instances. Also, we evaluated and reported a series of complex experiments that involve standard reinforcement learning elements—a serializable state and action representation, a world representation that is easy to explain and to understand but difficult to master, and a decision-making process that is not trivial. Moreover, differently from existing environments, we implement the Chef’s Hat competitive scenario, which is designed to enable data collection related to human play strategies and therefore to facilitate the modeling of online interactions between the agents. Future development will make the resulting model unable to define the playing strategies of a social robot.

Because learning the rules of a game, the Chef’s Hat game in particular, is not enough to generate competitive decision-making for artificial agents (Bai and Jin, 2020), learning heuristics of how the opponents play the game have a strong impact on the effectiveness of the learning agents. Allowing the agents to learn from a well-structured scenario and to adapt to continuous interactions to master how to play against other agents is one of the most significant contributions of our proposed simulation environment.

### 7.2 Chef’s Hat Online

The web interface of the Chef’s Hat simulation allows a hassle-free setup to include human-in-the-loop experiments. The capability to play the same game using the same structure, as in the simulated environment, gives us a powerful tool to extract heuristics from human behavior and directly compare it with data from the simulation environment.

In our experiments, the Chef’s Hat online implementation allowed us to demonstrate that our agents, although very effective when playing against each other, are still not a match for humans. The development of agents capable of playing against humans will be achieved when we start tackling the competitive aspects of Chef’s Hat, in particular by understanding and modeling how humans play the game. We believe that using the Chef’s Hat Online tool to evaluate different aspects of human–agent interaction will endow the development of much more complex and effective agents.

### 7.3 The Chef’s Hat Players Club

Yet another contribution to the community of online reinforcement learning is the implementation of 12 different types of learning agents for the Chef’s Hat game. The Chef’s Hat Players Club is presented as an established collection of players, with an associated benchmark and behavioral analysis, that can serve as the basis for the future development of online reinforcement learning. Agents that are developed following the Players Club structure can take part in the Chef’s Hat simulation environment, which will facilitate the development, assessment, and reproducibility of more complex algorithms. The integration of the Players Club with the Chef’s Hat Online interface also allows for the fast development of novel agents that leverage human data.

### 7.4 We Dig for COPPER, We Found Gold!

COPPER is a simple, yet efficient, solution for online and competitive learning based on Chef’s Hat. It has, however, the following limitation: as it is based on the experience replay, the more games an agent plays, the more examples are collected on the experience replay.

Our experiments gave us the insight that COPPER will stabilize the learning of the agent at some point, which makes it more robust against known opponents. If the COPPER-based agent plays against a large number of players, in theory, it would be able to generalize and play against several different types of opponents.

The main advantage of COPPER compared to the established PER algorithm is the speed at which the adaptation to specific players happens. All of our experiments demonstrated this characteristic, including when playing against the most complex agents of all—humans.

### 7.5 Limitations

The RL agents, especially in multiplayer settings, are susceptible to large variances in performance, depending on conditions. Therefore, although COPPER has a faster adaptation than PER in the scenario with different opponents and the COPPER-based agent achieved the best average victories when compared to the other agents in our experiments, performance variance is not considered and more data gathering and statistical validation will be needed in the future.

## 8 Conclusions

In this article, we introduce COPPER, a competitive experience replay algorithm for online reinforcement learning. To be able to better explain, assess, and provide our solution to the research community, we also propose a series of tools as follows: a novel simulation environment that implements a card game (Chef’s Hat) in a 1:1 manner; the Chef’s Hat Online interface that allows assessment and data collection from agents playing against humans; and the Chef’s Hat Players Club, a collection of implemented, trained, and benchmarked agents in the Chef’s Hat game.

Chef’s Hat was shown to be a plausible candidate for multi-agent and dynamic competitive interactions. We run an extensive number of experiments and demonstrate that our COPPER-based agents learn faster and more effectively than off-line and experience replay–based online agents.

Although the holistic analysis of COPPER demonstrates that its simple architecture learns how to adapt to different opponents playing Chef’s Hat over time, it still relies on several collected samples. We believe that investigating different opponent prediction mechanisms could help even further in the development of next-level competitive learning agents. In this study, we scratched the surface of an investigation of the behavior and performance of the COPPER-based agents against human players. Exploring this side of the work and understanding the modeling of dynamic strategies from humans is important future work.

## Data Availability

The datasets presented in this study can be found in online repositories. The names of the repository/repositories and accession number(s) can be found in the article/Supplementary Material.

## Footnotes

1
https://github.com/pablovin/ChefsHatGYM

2
https://github.com/pablovin/ChefsHatOnline

3
https://github.com/pablovin/ChefsHatPlayersClub
