# Supplementary material for: You Were Always on My Mind: Introducing Chef’s Hat and COPPER for Personalized Reinforcement Learning
Source: Front Robot AI. 2021 Jul 16;8:669990. doi: 10.3389/frobt.2021.669990 (PMC8323774; doi:10.3389/frobt.2021.669990)
Supplement: Supplementary file 1 [file DataSheet1.PDF]

Supplementary Material for You Were Always on My Mind: Introducing Chef´s Hat and COPPER for Continual Reinforcement Learning

1 CHEF´S HAT PLAYER CLUB OPTMIZATION

All of our agents were fine-tuned using the Hyperopt library **?**. We run 1000 evaluations per agent, and use the one with the best performance for all experiments. Performance is measured on the number of combined victories when playing 1000 games against 500 random agents, and 500 simple strategy agents.

1.1 Parameters Search Space

We run one exploration search per scenario, each of them with the search spaces defined in Table S1 for all the agents.

Parameter	Search Space
**DQL**	
Number of Layers	[1,2,3]
Units Per Layer	[16, 32, 64, 128, 256, 512, 1024]
Gamma	[0.5; 0.99]
Learning Rate	[0.0005; 0.9]
Double Q Learning	[True, False]
Target Net. Updates	[10, 50, 100, 250, 500, 1000]
**PPO**	
Number of Layers	[1,2,3]
Units Per Layer	[16, 32, 64, 128, 256, 512, 1024]
Gamma	[0.5; 0.99]
Learning Rate	[0.0005; 0.9]
Entropy Coeff.	[0.001; 0.9]
Value Func. Coeff.	[0.1; 0.9]
**ACER**	
Number of Layers	[1,2,3]
Units Per Layer	[16, 32, 64, 128, 256, 512, 1024]
Gamma	[0.5; 0.99]
Learning Rate	[0.0005; 0.5]
Entropy Coefficient	[0.001; 0.9]
buffer$_{size}$	[100, 500, 1000, 3000, 5000, 10000]

Table S1. Search space used to optimize all of our agents.

1.2 Final Architecture Opponents

Table S2 displays the final architecture used for both scenario for all of our agents.

The performance of each agent was very similar to each other, as exhibited in Figure S1, with the PPO agent reaching the maximum number of 956.2 victories.

**Parameter**	
**DQL**	
Number of Layers	2
Units Per Layer	32, 256
Gamma	0.98
Learning Rate	0.004
Double Q Learning	True
Target Network update	500
**PPO**	
Number of Layers	2
Units Per Layer	32, 256
Gamma	0.99
Learning Rate	0.05
Entropy Coeff.	0.008
Value Func. Coeff.	0.5
**ACER**	
Number of Layers	2
Units Per Layer	32, 256
Gamma	0.98
Learning Rate	0.003]
Entropy Coeff.	0.5
buffer Size	5000

Table S2. Final architecture for all of our agents.

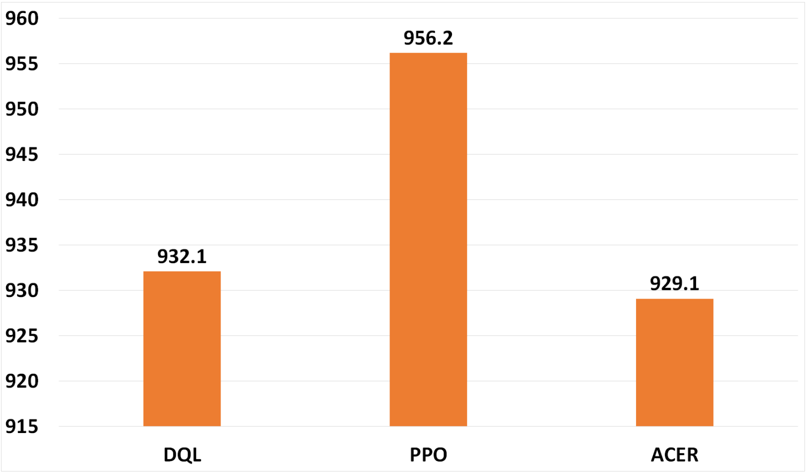

Figure S1. Total victories of the best agent of each type after the hyper-parameter selection.
